# Advances and future trends in real-time precision optical control of chemical processes in live cells

**DOI:** 10.1038/s44303-025-00083-1

**Published:** 2025-05-28

**Authors:** Chi Zhang, Bin Dong, Shivam Mahapatra, Seohee Ma

**Affiliations:** 1 James Tarpo Jr. and Margaret Tarpo Department of Chemistry, Purdue University, 560 Oval Dr., West Lafayette, IN 47907 USA; 2 Purdue Institute for Cancer Research, 201 S. University St., West Lafayette, IN 47907 USA; 3 Purdue Institute of Inflammation, Immunology, and Infectious Disease, 207 S. Martin Jischke Dr., West Lafayette, IN 47907 USA

**Keywords:** Imaging studies, Optical imaging, Imaging and sensing

## Abstract

Traditional chemical interventions regulate cellular processes but often affect non-target biomolecules. Precise and site-specific control is crucial for studying complex systems. Conventional laser-based methods offer high spatial precision and speed but rely on prior sample knowledge and do not apply to highly mobile targets. Real-time precision opto-control (RPOC) overcomes these limits using closed-loop feedback for automated and signal-determined real-time laser activation to regulate chemical processes in live biological samples. This review compares RPOC with other optical control techniques and explores its advancements, applications, and future directions.

## Introduction

Optical microscopy has long been at the forefront of biological research, providing scientists with the means to explore intricate living organisms at the cellular and molecular levels. From early simple lenses to modern advanced imaging systems, the field of optical microscopy has continuously evolved, offering insights into the structure, function, and dynamics of biological systems. Over the past few decades, advancements in super-resolution fluorescence microscopy have significantly improved spatial resolution^1–5^. The advancement in high-speed cameras and new imaging configurations such as light-sheet microscopy boosted the speed of fluorescence imaging^6–8^. Beyond resolution and imaging speed enhancements, chemical imaging techniques such as fluorescence microscopy^9^, Raman scattering microscopy^10,11^, and infrared microscopy^12,13^ have emerged as powerful tools in biological research. These techniques allow scientists to visualize and analyze the spatial distribution of molecules within biological samples, enabling the study of complex biological processes such as protein interactions, cellular signaling, and metabolic fluxes with high spatial resolution and sensitivity. Optical microscopy techniques, especially fluorescence microscopy, have now permeated nearly all aspects of biological science. However, microscopy itself, as a purely visualization-based approach, provides no more than passive observation of the systems under study.

In many cases, simply monitoring chemical processes in living samples passively is insufficient. To better understand the relationships between different biological processes, it is essential to control these processes while simultaneously monitoring sample responses. Traditionally, this control has been achieved through chemical interventions, such as treating cells with compounds. This approach assumes that the compounds interact selectively with only the desired targets. However, molecular interactions are not spatially confined, and introduced compounds can interact with both desired and undesired molecules and pathways, leading to off-target effects that complicate the results^14–17^.

Optical control methods have revolutionized the study of biological systems by enabling researchers to manipulate and control cellular processes with high spatial precision. Intense photons can exert mechanical forces to manipulate microscopic objects through optical tweezing effects^18–20^. However, optical tweezers are primarily designed for the physical and mechanical control of cells. In contrast, photons can also enable chemical control through various photochemical processes, such as photobleaching^21,22^, photouncaging^23,24^, photoactivation^25,26^, and photocatalysis^27–29^. These processes allow input photons to modify chemical structures or drive reactions, either globally throughout the sample or locally at specific sites of interest. For example, precise laser delivery to subcellular compartments enables dynamic studies of proteins and organelles using fluorescence loss in photobleaching (FLIP) and fluorescence recovery after photobleaching (FRAP) techniques^30–32^. Photouncaging of small molecules or activation of complex compounds can precisely release active chemical species to specific targets^23,24,33–35^. In neuroscience, photoactivation of proteins has been widely used to control neuronal activities^36,37^. Additionally, the concept of photocatalysis has expanded beyond synthetic chemistry to regulate chemical processes, particularly redox reactions, in live cells^27–29^. Furthermore, femtosecond (fs) laser treatment has opened new frontiers in microsurgery and targeted therapy. The ultrafast and nonlinear nature of fs pulses enables highly localized tissue ablation with minimal thermal damage, making it a powerful tool for applications such as neuronal microsurgery^38,39^, intracellular surgery^40,41^, and light-controlled genetic activities^42,43^. These methods open new possibilities for studying and controlling living systems, providing a level of control and precision that traditional chemical interventions cannot achieve.

Furthermore, recent advancements in patterned illumination (PI) techniques have further enhanced optical control, particularly in neuroscience, where researchers can use holographic light patterns to precisely stimulate or inhibit neuronal activity. By dynamically shaping laser beams, PI-based optogenetic techniques allow the activation of specific neurons within intricate neural networks, enabling studies of functional connectivity and circuit dynamics with unprecedented precision^37,44,45^.

Despite advancements in optical control technologies, these methods require prior knowledge of the sample and have difficulties in selectively targeting highly mobile molecular entities in living systems. Recently, a new opto-control approach integrating fast laser scanning with real-time signal processing and laser activation, termed real-time precision opto-control (RPOC), was developed^46–48^. This technology utilizes chemical-specific optical signals from the sample to trigger another action laser that selectively controls chemical processes associated with desired chemical targets. Signal detection, processing, decision-making, and laser activation occur in real time within the same pixel with a response time at the nanosecond level. This enables simultaneous tracking and control of molecular targets with high spatial precision, even for those with high mobility. The target selection is automated and optical-signal-determined. In this review, we summarize the principles, recent technological advancements, and current applications of RPOC. We also compare RPOC with other optical control schemes used in biological science. Additionally, we offer insights into the future directions of RPOC, considering both technological advancements and potential applications.

## Conventional optical methods for chemical control in biological samples

### Nonspecific illumination

The earliest and most conventional method of using light to drive chemical changes in biological systems involves direct, non-specific illumination of the sample. In this approach, light is applied globally, and the chemical reaction is governed not by spatial control of illumination but by the distribution of photosensitive species within the sample. This method is widely applicable when uniform activation of photosensitive compounds is desired or when the compound exhibits a strong affinity for the target of interest. In photodynamic therapy^49^, patients are typically treated with compounds that accumulate at the diseased sites, followed by global light illumination. This approach of global sample illumination was also initially used to control neural activities in optogenetics^36,50,51^. Optogenetics is a groundbreaking technique that enables precise optical control of cellular activities, particularly in neuroscience. By genetically encoding light-sensitive proteins, such as channelrhodopsins and halorhodopsins, researchers can manipulate neuronal activity with high spatial and temporal precision^36,52^. This technology has profoundly impacted neurobiology, circuit mapping, and even therapeutic interventions for neurological disorders. Global illumination has been used to stimulate neurons with light-sensitive opsins. Furthermore, nonspecific light illumination has been applied in low-level laser therapy (LLLT) for wound healing^53^, tissue regeneration^54^, photothermal therapy^55^, pain management^56^, and anti-inflammatory effects^57^. Light radiation has been reported to regulate ATP production^58^, the NF-κB pathway^59^, and antioxidant activity^60^, thereby offering therapeutic benefits.

### Point scanning

Photons in the nonspecific illumination scheme do not provide spatial specificity. Several technical approaches can achieve spatially selective optical manipulation with high precision. One common method is point scanning of lasers, which uses galvanometer-driven mirrors to rapidly guide a focused laser beam across the sample^61^. This technique is typically integrated with laser scanning microscopes to visualize post-treatment effects. The point-scanning method offers the advantage of delivering high laser intensity at the focal point with exceptional spatial precision. Its integration with confocal or multiphoton fluorescence techniques further enables high-precision 3D laser positioning and imaging. This approach is particularly beneficial for controlling and imaging targets with 3D distributions or those located in deeper tissue with high spatial precision^62^. Additionally, it exhibits a high tolerance for laser beam quality and can be easily adapted to a broad wavelength range. However, compared to the PI method, point scanning is generally slower and cannot simultaneously address multiple targets within the field of view (FOV). This limitation makes it suitable for studying relatively slow biological processes and scenarios that do not require simultaneous control of multiple targets. Furthermore, in a conventional opto-control system using point scanning, the optical treatment and imaging processes are separated, creating a time gap that often prevents the monitoring of molecular changes in real time during optical treatment.

**Fig. 1:**
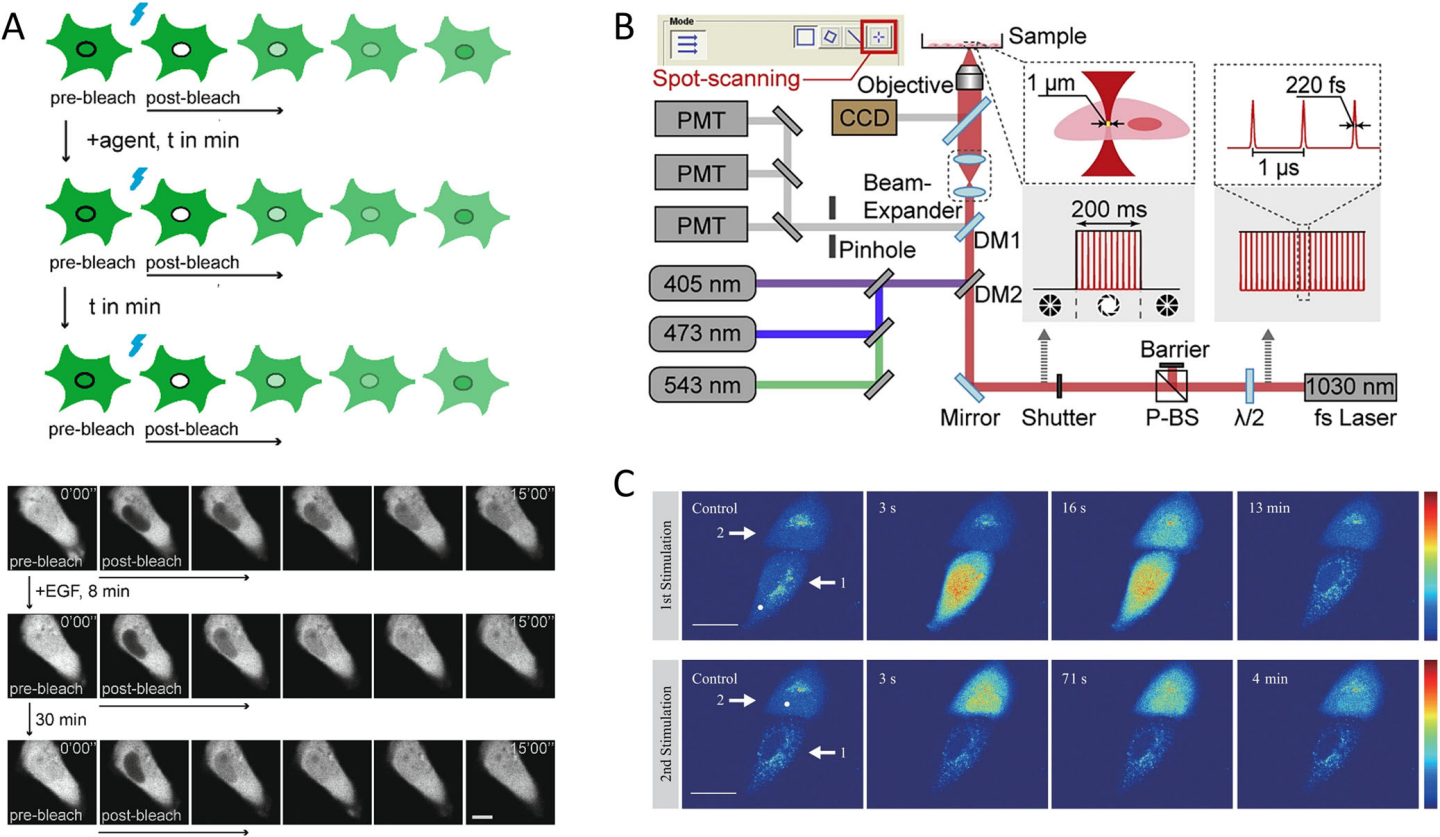
Point-scanning methods for opto-control. **A** (Top) Schematic of repeated fluorescence recovery after photobleaching (FRAP) analysis of CapG in the cell nucleus of the same live breast cancer cell, serving as a functional readout for intracellular protein dynamics. (Bottom) An MDA-MB-231 cell showed increased nuclear CapG-GFP import following EGF addition, as observed through repeated FRAP. FRAP experiments were conducted before EGF addition and at 8 min and 30 min post-treatment. Scale bar: 10 µm. Figures adapted from ref. ^63^. **B** Optical setup for femtosecond laser (1030 nm, ~220 fs, 1 MHz) photoactivation. The laser is focused on a submicron region using a 60× objective, with exposure duration controlled at 200 ms by a mechanical shutter. Figure adapted from ref. ^64^. **C** Representative time-lapse images of laser-induced Ca²^+^ response and cell death in HeLa cells. The white spot marks the irradiated region. Time stamps above each image indicate the elapsed time after laser stimulation at 0 s (control). Scale bar: 20 µm. Figure adapted from ref. ^65^.

Confocal microscopes typically feature galvo mirror control for directing laser light to manually outlined regions, enabling targeted interactions. This functionality is commonly used for fluorescence studies such as FRAP and FLIP, allowing researchers to investigate protein and organelle dynamics in live cells. Figure 1A illustrates a methodology developed for repeated FRAP analysis of the actin-binding protein CapG, where photobleaching of fluorophores in the cell nucleus was performed^63^. The study demonstrated that upon epidermal growth factor (EGF) addition, some MDA-MB-231 cells exhibited increased nuclear transport after 30–50 min, suggesting that EGF may enhance CapG nuclear shuttling^63^. However, due to the separation between photobleaching and imaging, real-time protein dynamics during photobleaching were not captured.

Figure 1B presents an optical schematic that employs a fs laser for photoactivation. The fs laser pulses (1030 nm, 220 fs, 1 MHz) are focused to ~1 µm and controlled by a mechanical shutter with a 200 ms exposure time^64^. This method has been shown to enable the noninvasive activation of stem cells, including human umbilical cord mesenchymal stem cells (hUC-MSCs) and granule neuron progenitors (GNPs), promoting differentiation both in vitro and in vivo.

Figure 1C shows another study where high-power fs laser pulses were directed at the endoplasmic reticulum (ER), triggering a Ca^2+^ spike release^65^. This interaction not only induced dynamic Ca^2+^ responses in the targeted cell but also affected neighboring cells. However, subsequent stimulation of the neighboring cells did not elicit the same propagation, indicating that fs laser pulses above a certain threshold can cause irreversible cytotoxic effects in irradiated cells, rendering them unresponsive to further laser stimulation.

The above examples highlight directing lasers for precise opto-control in subcellular locations using point-scanning platforms. Despite being used in various applications, conventional galvo-based methods for controlling biological processes remain rudimentary. Typically, an image is acquired to guide decision-making, followed by the manual selection of targets on the sample, which are then treated with specific lasers prior to imaging the cellular response. This approach is inherently limited for precise control of highly mobile targets, as the target locations continuously change. Additionally, accurately selecting and outlining molecular targets with complex shapes could be difficult or impractical. Moreover, because optical perturbation and imaging are usually separated in this workflow, crucial real-time cell response information is lost during the opto-control process.

### Patterned illumination (PI)

Another widely used approach, particularly in neuroscience, is PI, which enables spatially selective excitation or inhibition of biological targets. Typically, an image is first acquired to identify targets of interest. A computer-generated holographic (CGH) mask is then applied to convert a wide laser beam into the desired light pattern, concentrating most of the laser intensity on the targets^52,66,67^. Common devices for spatial light modulation include liquid crystal devices^68^, deformable mirrors^69,70^, and micro-electro-mechanical systems (MEMS)^71^. The key advantage of widefield PI is its ability to simultaneously and continuously illuminate multiple targets, making it particularly valuable in modern neuroscience for studying neural circuits. Its compatibility with high-speed cameras enables the rapid capture of cellular responses with minimized phototoxicity. On the other hand, compared to the point-scanning method, PI generally provides lower spatial resolution and lacks 3D sectioning capabilities. Additionally, CGH optimization is typically wavelength-specific, resulting in a lower tolerance for laser beam quality and wavelength changes compared to point scanning. The absence of a confocal configuration further limits 3D sectioning capability and often leads to reduced image quality^61,62^.

PI methods have been widely used when simultaneous illumination of various spots is needed, especially in optogenetics. Compared to traditional optical control methods such as photopharmacology and two-photon uncaging, optogenetics offers unmatched specificity and reversibility. PI techniques significantly enhance optogenetics by providing flexible spatial and temporal control, enabling more physiologically relevant and precise investigations into neural circuits and cellular functions. For example, using a configuration using a liquid-crystal spatial light modulator (SLM), Reutsky-Gefen et al. demonstrated that phase CGH can be created to generate various illumination patterns onto retinal nerve cells after field lens and objective lens projection^52^. Microelectrode arrays were used to record responses of channelrhodopsin-2 (ChR2)-expressing retinal neurons. This study successfully uses holography to precisely stimulate retinal ganglion cells in blind retinas, achieving cellular resolution control with millisecond temporal precision. This approach allows flexible and spatially controlled activation of distributed neuronal circuits, which is essential for generating meaningful visual perceptions in the brain^52^.

Automated feedback control for precise signaling input regulation in live cells has been developed, using light-gated protein interactions to perturb protein localization and phosphoinositide 3-kinase (PI3K) activity while buffering against variability^72^. The feedback loop demonstrated a frame-by-frame response with a time scale of seconds^72^.

**Fig. 2:**
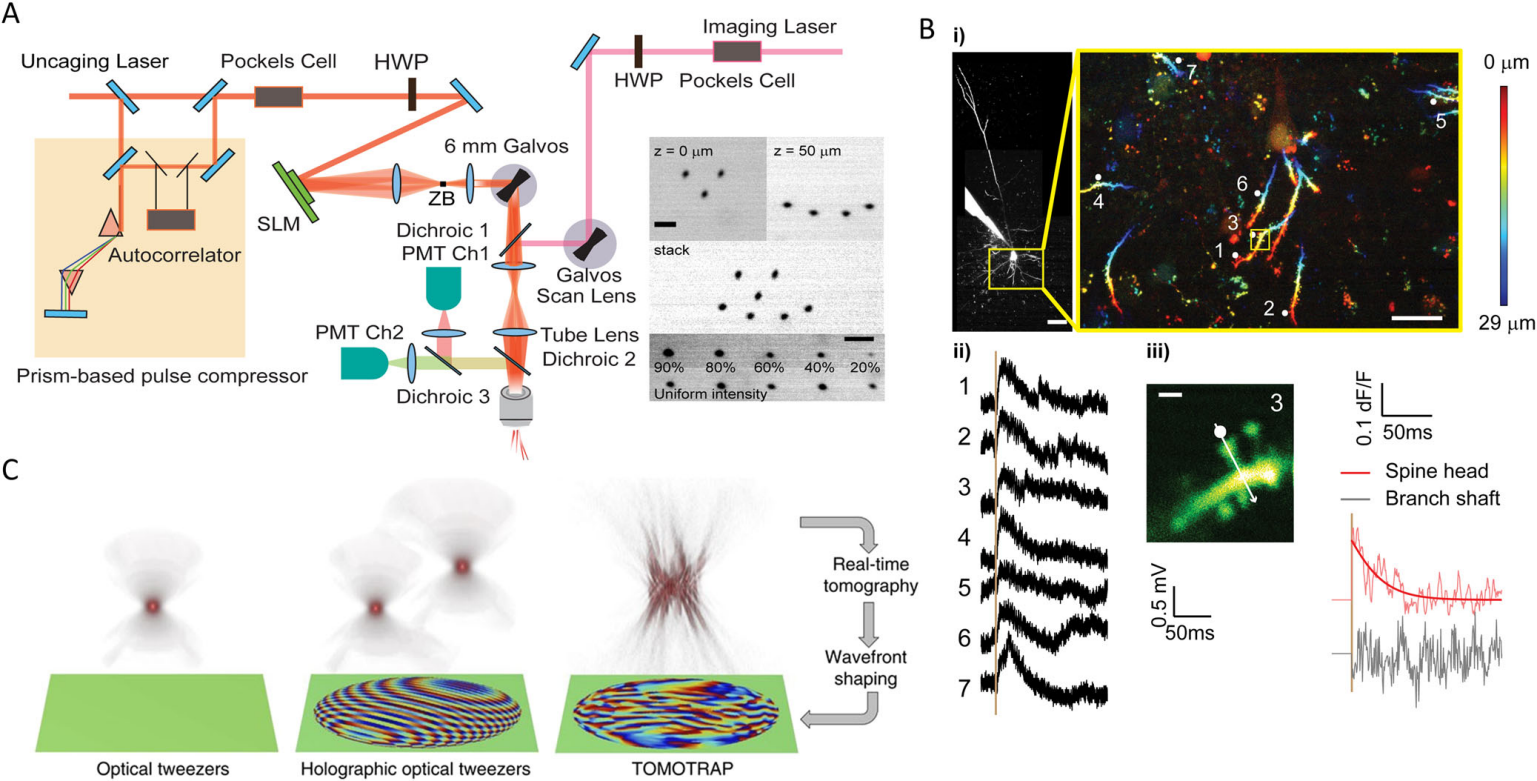
Patterned illumination methods for opto-control. **A** The two-photon microscope features both imaging and uncaging pathways. (Right inset) A 3D holographic pattern is shown (top), along with independently adjustable power weighting for each holographic spot (bottom). Scale bars, 5 μm. Figure adapted from ref. ^73^. **B** 3D two-photon SLM-based glutamate uncaging: (i) Example of a Layer 5 pyramidal neuron with a magnified view of its basal dendrites. Scale bars: 50 μm (main) and 20 μm (inset). (ii) Scanless single-spine excitatory postsynaptic potentials (EPSPs) recorded from seven spatially distributed basal dendritic spines. (iii) Calcium line-scan across spine #3 (yellow square in i). Scale bar: 1 μm. Figure adapted from ref. ^73^. **C** Schematic representations of various optical tweezers: From left to right—Single-beam optical tweezers, holographic optical tweezers, and the tomographic mold for optical trapping (TOMOTRAP), which integrates real-time 3D refractive index tomography with laser wavefront shaping. The top row illustrates the 3D beam intensity distributions generated by each optical tweezer configuration, while the bottom row displays the phase components of the complex optical field of the trapping beam. Figure adapted from ref. ^78^.

PI methods can also control sites at the subcellular level, such as neural synapses. As shown in Fig. 2A, B, the authors combined two-photon imaging with 3D holographic glutamate uncaging at synapses^73^. To capture 3D depth information, the SLM-based PI system was integrated with high-speed laser scanning for two-photon imaging. Here, scanless holographic uncaging was performed across multiple synapses within a 100 μm × 100 μm × 35 μm field of FOV, demonstrating consistent efficiency and specificity at each site. Calcium imaging confirmed localized signaling within spine heads, with minimal off-target activation in dendritic shafts^73^.

The PI method and automated feedback control have also been applied to advanced optical tweezing^74–77^. Figure 2C compares three optical trapping methods: single-beam optical tweezers, holographic optical tweezers, and the advanced tomographic mold for optical trapping (TOMOTRAP)^78^. TOMOTRAP measures 2D refractive index maps of the sample in real-time and uses SLM to generate a 3D light field distribution based on the 3D refractive index map. This enables continuous updates to the optical forces, allowing precise control over the orientation and movement of complex particles.

Despite having wide applications in various fields, PI methods still require prior knowledge of the sample, limiting their applicability to highly mobile targets. Target selection is typically user-driven rather than automatically guided by the sample’s chemical distribution. This manual tracing of chemical features, particularly those with high mobility or complex distribution, can be impractical. Furthermore, although PI has been demonstrated to work with multiphoton fluorescence^79^, it is not the most optimal method. As a wide-field illumination method, PI is largely incompatible with other nonlinear chemical imaging techniques that rely on tight laser focusing, such as stimulated emission depletion (STED) microscopy, coherent Raman scattering (CRS), harmonic generation, and transient absorption.

## Real-time precision opto-control (RPOC)

Both galvo scanning and PI face significant challenges in real-time, automated, and high-precision opto-control, especially for dynamic molecular targets in living systems. These methods rely on pre-acquired images and predefined targeting strategies, making it challenging to track and control targets that exhibit rapid, unpredictable motion. Feedback-control mechanisms have been implemented in PI methods for optical tweezers and optogenetics on a frame-by-frame basis, with a response time on a level of seconds to milliseconds^72,76,78^. In point scanning, fast feedback has been applied in fluorescence imaging to reduce phototoxicity. For example, controlled light-exposure microscopy uses a real-time feedback system to regulate the exposure time at each pixel^80,81^. Adaptive illumination with microsecond response time has also been utilized in STED microscopy to minimize the impact of the stimulated emission laser on biological functions and prevent photobleaching^82,83^. However, these approaches are primarily tailored for improving imaging quality, rather than for controlling chemical processes in cells. An optical system designed to precisely control chemical species in live cells or organisms requires selective chemical detection, real-time feedback control on the microsecond to nanosecond scale, high-speed decision-making, a wide range of lasers to regulate chemical processes, and sensitive readouts to evaluate the changes induced by optical control. These capabilities must be optimally and dynamically integrated within a laser scanning platform.

To overcome these limitations, we developed RPOC as a new approach. RPOC is based on high-speed point-scanning and therefore is easily adapted to all laser scanning configurations. It boasts a response time in the tens to hundreds of nanoseconds, enabling decision-making within a single pixel. In RPOC, the laser interaction sites are automatically determined by chemical-specific optical signals. The confocal or multiphoton configuration enables intrinsic 3D control and imaging capabilities, offering advantages for controlling and recording cellular responses in 3D and in deeper tissue layers. It also has a high tolerance to a broad range of laser wavelengths, which is critical for controlling chemical processes using both single-photon and multi-photon processes under the same conditions.

**Fig. 3:**
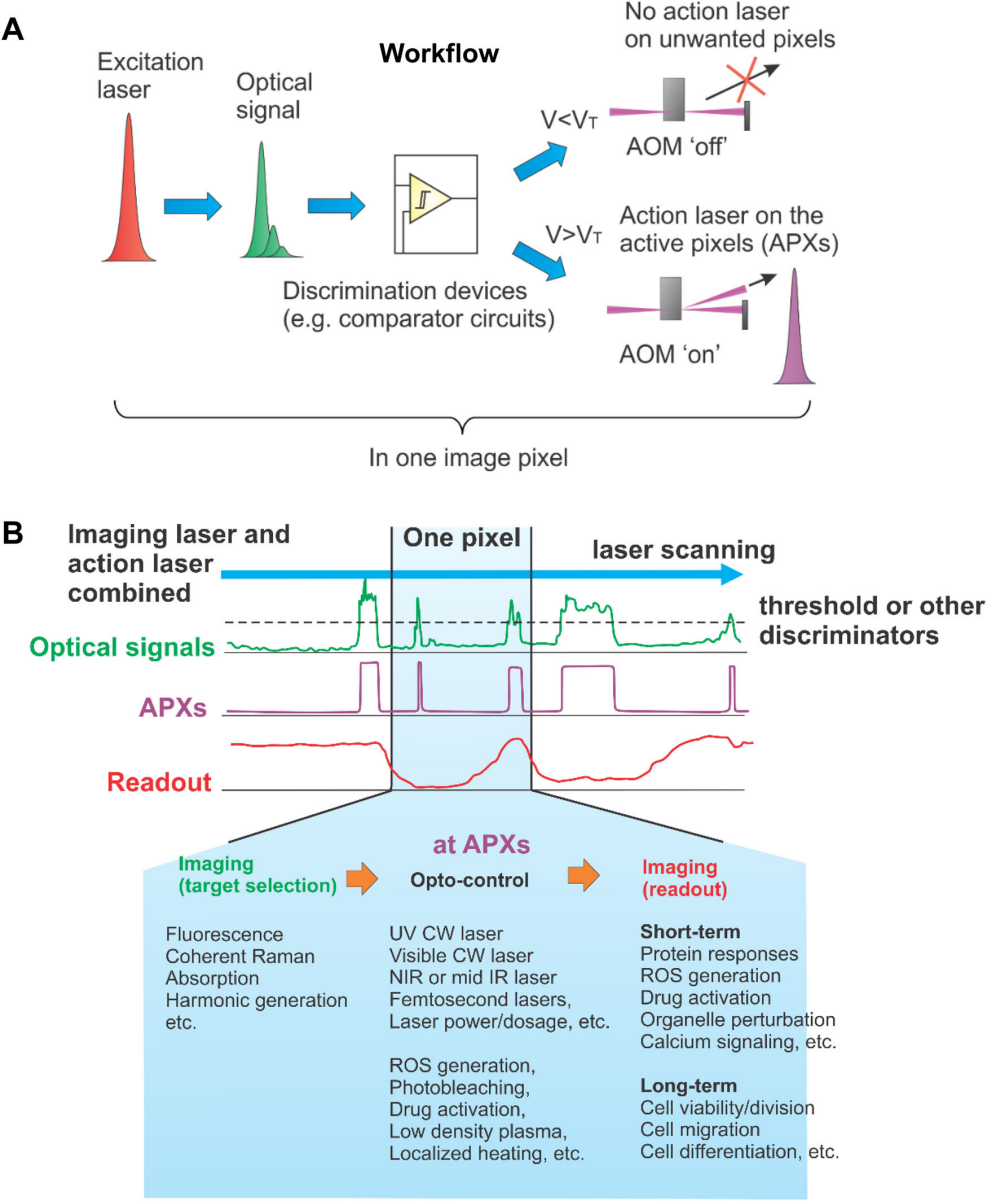
Real-time precision opto-control (RPOC). **A** Workflow of a typical RPOC system, illustrating the integration of imaging, opto-control, and real-time feedback. AOM acousto-optic modulator. V_T_ is the voltage threshold for optical signals. **B** Schematic representation of real-time processes at any pixel during RPOC, including target selection, opto-control, and readout. Listed are various imaging modalities for target selection, laser options and mechanisms for opto-control, and key features for readout. APX active pixels, ROS reactive oxygen species, CW continuous wave, NIR near-infrared, UV ultraviolet.

The workflow of RPOC is illustrated in Fig. 3A. The excitation laser continuously scans the sample, generating optical signals that are sent to a discrimination (or decision-making) device. For example, a comparator circuit can be used to set a voltage threshold and continuously compare the optical signal with it during laser scanning^46^. If the optical signal voltage exceeds the threshold voltage, the comparator circuit outputs a transistor-transistor logic (TTL) 1; otherwise, it maintains a TTL 0. These TTL signals control a fast light shutter such as an acousto-optic modulator (AOM), enabling rapid activation or termination of the action laser^46,48^. Once activated, the action laser is collinearly combined with the excitation laser, ensuring precise opto-control at the exact pixels where the desired optical signals are detected. The system’s response time is typically in the range of tens to hundreds of nanoseconds, significantly faster than the pixel dwell time. This enables real-time operation without requiring prior knowledge of the sample and allows for automated, on-the-fly target selection.

Different from other opto-control technologies which usually require image acquisition followed by decision-making by the user, RPOC enabled automated and real-time laser control at only the pixels wanted. During laser scanning, three channels operate in parallel, as illustrated in Fig. 3B:

1. Target Selection – Identifies targets for opto-control – excitation laser

2. Opto-Control – Executes the opto-control of chemical processes – action laser

3. Readout – Monitors and records sample responses to opto-control – readout laser

The target selection channel generates optical signals that reveal chemical information from the sample, while the opto-control channel processes these signals in real time to determine the activation of the action laser. The pixels where the action laser is activated are referred to as active pixels (APXs). Simultaneously, imaging channels function as readouts, capturing both short-term and long-term responses of the sample. These processes are facilitated by various lasers, including excitation lasers, action lasers, and readout lasers. Depending on the specific application, different lasers can be selected for each function. In many cases, the same laser can serve both target selection and readout, and lasers designated for different purposes may sometimes be interchangeable.

### Target selection

For target selection, RPOC can utilize various chemical-selective optical processes, with fluorescence being the most effective due to its high sensitivity and chemical specificity. Fluorescence enables organelle selection using dyes and facilitates protein detection via fluorescent proteins. However, it also presents challenges, including photobleaching, which diminishes optical signals at APXs and leads to a gradual reduction of APXs throughout treatment. Additionally, laser illumination of organelle fluorescent dyes can induce photosensitization, causing significant cellular perturbation and potential toxicity^84^.

Alternatively, label-free chemical imaging methods, such as CRS, provide chemical contrast without requiring external labels^11,85^. However, due to the nature of bond-selective imaging and its limited sensitivity, CRS typically applies only to chemical compositions that are highly enriched in a specific chemical bond. In most biological samples, this condition is met by the accumulated neutral lipid in lipid droplets (LDs)^85,86^ and overall proteins^87,88^. Besides LDs and total proteins, CRS can sometimes provide good contrasts from exogenous molecules possessing distinct chemical bonds that give signals in the Raman silent region, such as accumulated drugs, carbon-deuterium bonds, or alkyne bonds^89–94^. Other chemical-selective imaging techniques, including infrared (IR) absorption, transient absorption, and harmonic generation, also offer certain degrees of chemical specificity but have not yet been explored for RPOC target selection.

### Opto-control

Various lasers can be selected as action lasers for controlling chemical processes in living samples. For example, UV light at 375-405 nm can directly induce reactive oxygen species (ROS) without the need for photosensitizers, serving as a direct laser perturbation to cells or subcellular compartments^48,95,96^. Such ROS generation usually involves the simultaneous presence of endogenous chromophores, oxygen, and photons. ROS such as singlet oxygen (^1^O_2_), superoxide anions (O_2_^−^•), hydrogen peroxide (H_2_O_2_), and hydroxyl radicals (•OH), can cause oxidative stress and cellular damage.

**Fig. 4:**
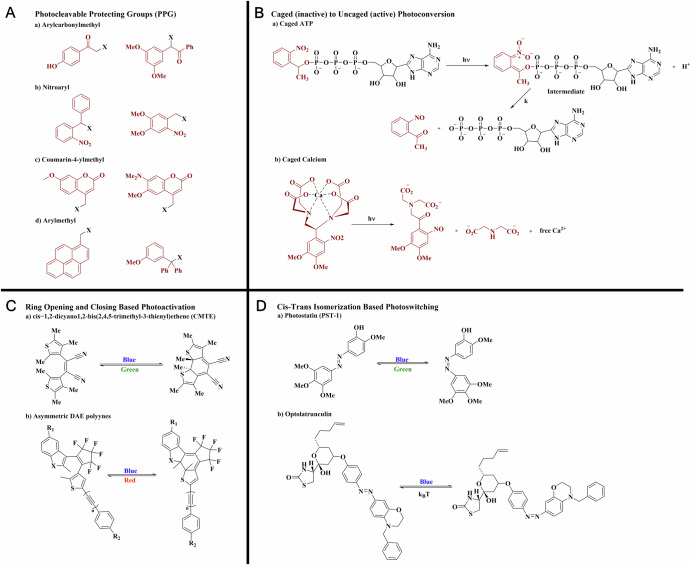
Light-controlled molecules and functional groups. **A** Examples of photocleavable protecting groups including arylcarbonylmethyl, nitroaryl, coumarin-4-ylmethyl, and arylmethyl groups. **B** Photoreactions of caged ATP and calcium, enabling controlled release upon light activations. **C** Photoswitchable molecules undergoing ring opening and closing upon light interactions. **D** Photoswitchable processes based on azobenzene *cis-trans* photoisomerization, enabling reversible structural changes under light exposure.

In addition, UV-blue light enables the photouncaging of various caged small molecules. The caged compounds are usually biologically inactive molecules protected by a photolabile group, that releases the active substance upon exposure to UV or blue lasers. While the initial delivery of these caged molecules is typically nonspecific, their active forms can be uncaged with high spatial precision using laser illumination. For example, In neuroscience, photouncaging is widely applied to neurotransmitters like glutamate to study synaptic transmission and neural circuit dynamics. In Fig. 2B, two-photon photouncaging of caged glutamate is used to activate neurons by releasing bioactive glutamate molecules specifically at synapses^73^. This method provides high spatial and temporal control over neural excitation, making it an essential tool in neuroscience research. For drug release applications, various photocleavable protecting groups (PPGs) can be used to temporarily mask the active compound (Fig. 4A), allowing its release upon light exposure when needed^97^. UV-blue light effectively facilitates the photorelease of various bioactive molecules such as ATP and calcium (Fig. 4B) from their protected forms, enabling precise control over biochemical and pharmacological processes^34,98–101^.

Additionally, the UV-blue wavelength range can alter the states of photochromic molecules and induce conformational changes of photoswitchable inhibitors^25,46^. The photon energy at the UV-blue range allows for the induction of many ring-opening processes (Fig. 4C)^102–105^ and twist-and-turn conformational changes of azobenzene-based compounds (Fig. 4D)^25,106^. Green lasers, typically between 500-550 nm, can be used as action lasers to pair with the UV-blue to reverse the structure changes of photochromic molecules or photoswitchable inhibitors induced by the 405 nm laser.

Additionally, both blue and green lasers, when used with RPOC, enable adaptive photobleaching^21,22^. This capability allows the automatic selection of photobleaching targets and concurrent photobleaching during imaging, thus enhancing the performance of FRAP and FLIP for better studies in protein dynamics, particularly for targets exhibiting complex patterns in the FOV^107^.

Besides visible continuous wave (CW) lasers, other light sources such as near-IR lasers or fs lasers can be applied as action lasers. The former would allow for selective sample heating, while the latter could perform site-specific molecular perturbation based on multiphoton absorption processes. The fs laser interaction with cellular compartments depends on both the average and peak power^108^. At high peak power, fs laser pulses can generate low-density plasma and ROS through multiphoton absorption^108^. Compared to CW lasers, fs-pulsed laser opto-control provides high spatiotemporal precision in both the lateral and axial directions^108^.

### Readout

The readout of sample responses post-opto-control is essential for understanding the effects of site- or chemical-specific optical manipulation. This is achieved by exciting samples with readout lasers and detecting the corresponding signals. These lasers and detection channels may be the same or different from those used for target selection. For example, a 589 nm laser can excite a red mitochondrial dye, while a 473 nm or 488 nm laser reads out changes in green fluorescent proteins^48^. Signals are typically detected simultaneously using separate detectors.

Readout responses are categorized as short-term or long-term based on duration. Examples of short-term responses (seconds to an hour) include dynamic protein movements (e.g., tubulin, actin, end-binding proteins), mitochondrial membrane potential changes, fluorescent protein oxidation, and ROS generation. Long-term responses (hours to days) examples involve cell viability, division, differentiation, migration, or embryo development. RPOC can analyze both short and long-term responses, with long-term studies requiring a stable sample microenvironment. Modern stage-top incubators help maintain optimal temperature, humidity, and CO_2_ levels, enabling continuous monitoring of the same or multiple FOVs to assess RPOC-induced changes.

## Instrumentations of RPOC

**Fig. 5:**
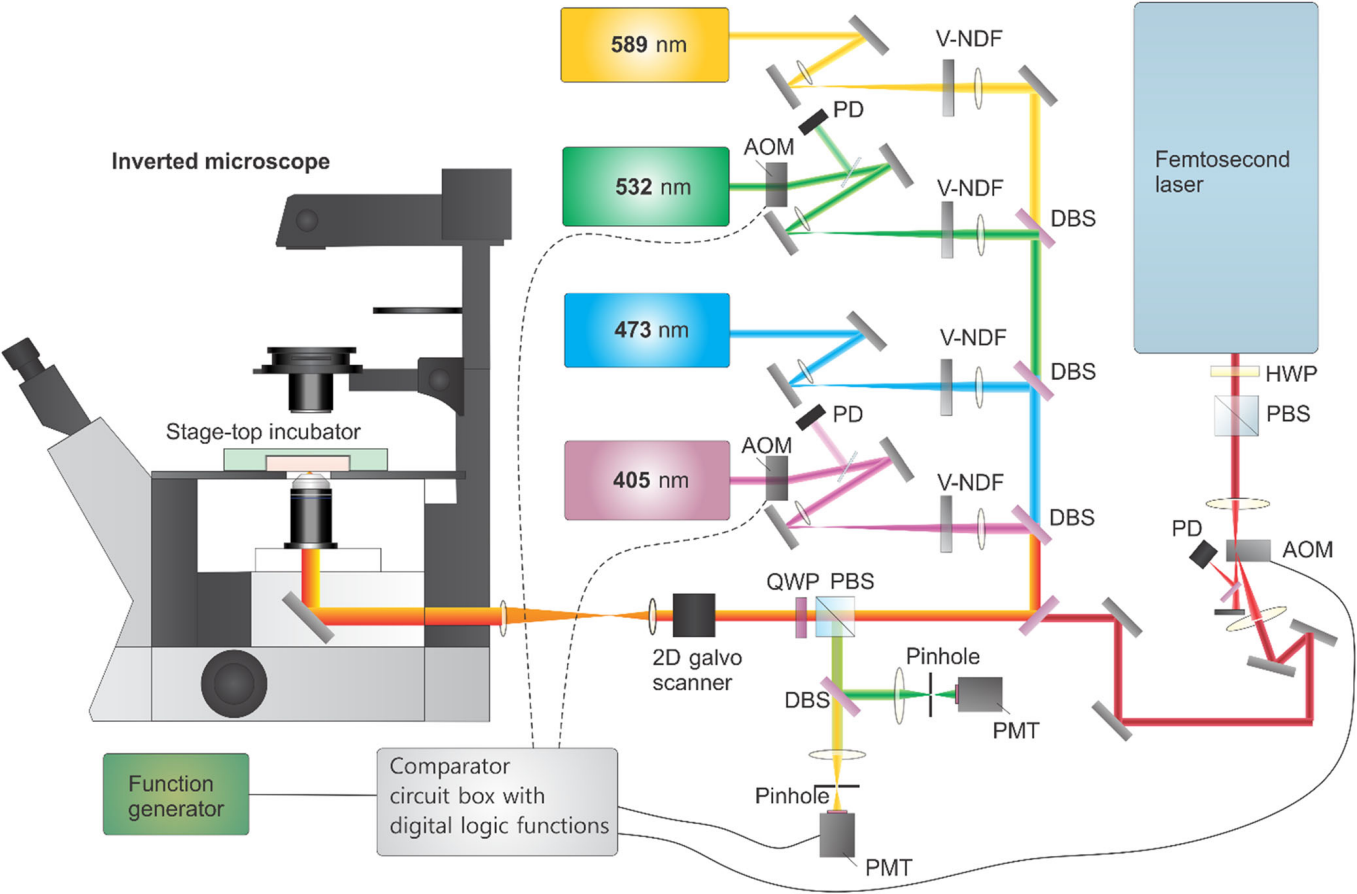
An example of optical and feedback control layout of RPOC. The schematic illustrates the optical and feedback control design of the RPOC system, with 405 nm and 532 nm CW lasers, and a femtosecond laser as options for action lasers. A comparator circuit enables real-time decision-making for RPOC, supporting simultaneous pulse-picking of the femtosecond laser when used with a function generator. AOM acousto-optic modulator, PD photodiode, DBS dichroic beam splitter, PBS polarization beam splitter, HWP half-wave plate, QWP quarter-wave plate, PMT photomultiplier tube, V-NDF variable neutral density filter. The PBS and QWP can be replaced with a multiband-pass beam splitter, and free-space lasers can be substituted with fiber-coupled lasers. Additionally, AOMs can be replaced by electro-optic modulators (EOMs) or direct laser control.

The key instrumentation components of RPOC include a laser scanning microscope, a fast decision-making system, and rapid switches for action lasers. The microscope typically has multiple lasers for excitation, action, and readout, as well as multiple detection channels for target selection and readout. A typical RPOC system is illustrated in Fig. 5. This system consists of four CW lasers: two for excitation and readout (589 nm and 473 nm) and two as action lasers (405 nm and 532 nm). AOMs are placed in the beam paths of the action lasers, allowing the first-order deflected beams to be coupled into the sample pixel along with the other laser beams.

APX signals can be recorded either by a photodiode or directly through the comparator circuit, which enables real-time comparison and computation of optical signals against a preset threshold or other discrimination criteria. Optical signals are detected by one or more photomultiplier tubes (PMTs). Laser power is controlled using variable neutral density filters placed in each beam path or directly using analog modulators of the lasers.

In Fig. 5, a fs laser is also shown as an optional action laser for interacting with biological samples via laser pulses. Like other action lasers, fs laser activation is controlled by an AOM. To enable RPOC with pulse-picking for separate control of both average and peak power, a function generator can be used in conjunction with the comparator circuit^108^.

High-speed 2D galvo mirrors scan the lasers across the sample, which is housed within a stage-top incubator. This incubator precisely regulates temperature, humidity, and CO_2_ levels to maintain optimal conditions for live cells. The stable microenvironment is critical for obtaining long-term cellular responses following RPOC.

Aside from relying solely on the comparator circuit, a recently developed RPOC software enables precise narrowing of the region of interest (ROI) for opto-control^107^. This software-assisted RPOC system allows molecular targets to be controlled using different color lasers and varying dosages across multiple ROIs within the same FOV, greatly enhancing the flexibility and throughput of RPOC.

The spatial precision of RPOC is diffraction-limited, typically ranging from 300 to 500 nm in the lateral dimension. The response time of RPOC at each pixel, when AOMs are used, is approximately 500 ns, primarily constrained by the response time of the AOM driver^48^. Alternatively, when action lasers are directly controlled by the comparator circuit, the response time can be reduced to <100 ns. The maximum laser scanning speed is primarily determined by the galvo mirror hardware, typically ranging from 1 to 7 kHz. If resonance mirrors or polygonal mirrors are used, the laser scanning speed can be further increased to tens of kHz.

## Applications

### Highly precise microsurgery with lasers

**Fig. 6:**
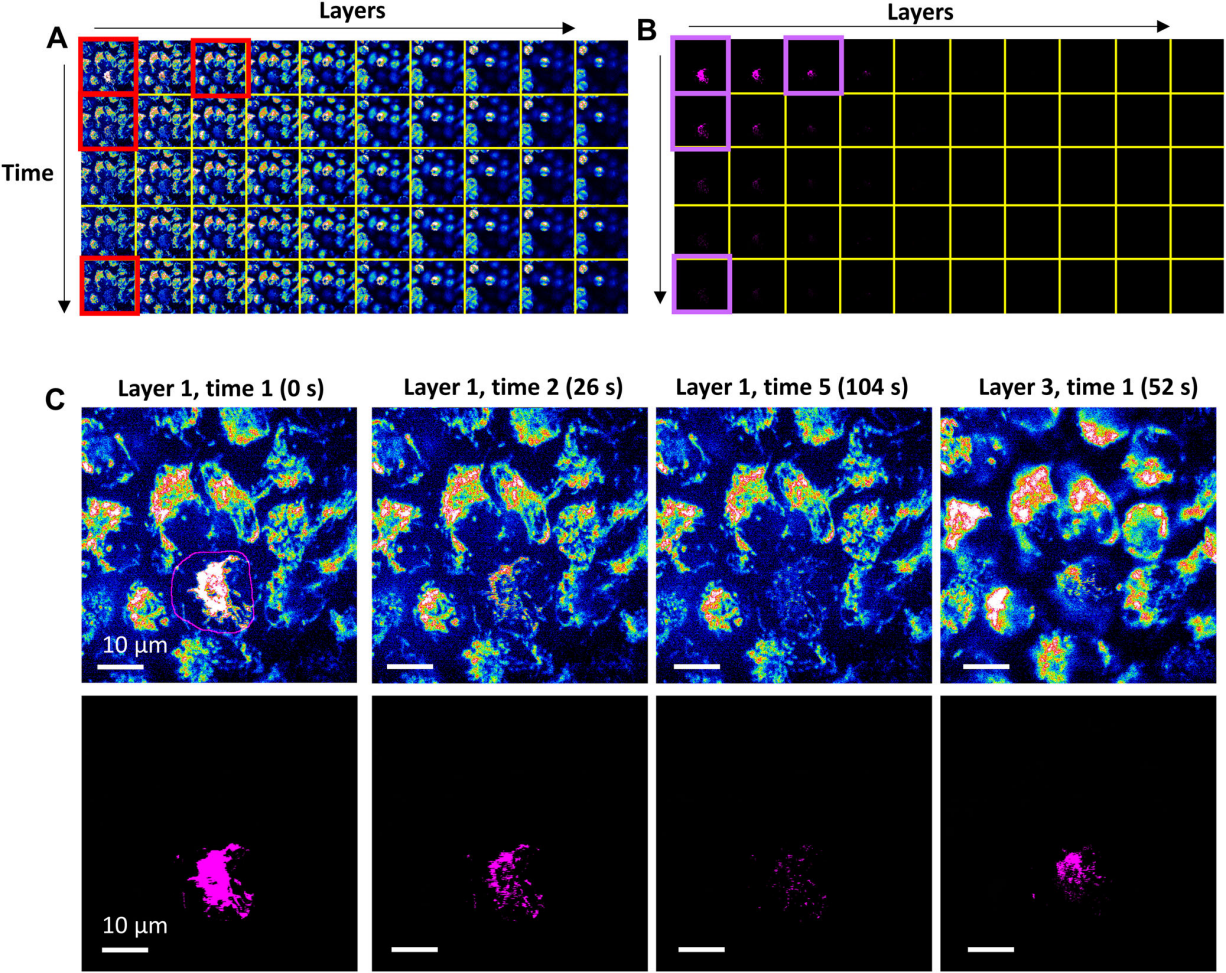
Precise 3D microsurgery of mitochondria in live cells. **A** MitoTracker fluorescence signals (Jet color scheme) collected in 3D over time during RPOC treatment. **B** APX signals from the corresponding layers of a selected cell in panel (**A**). The action laser is a 1045 nm femtosecond laser with 150 fs pulse width (~250 fs on the sample), 80 MHz repetition rate, and 22 mW average power. The pixel dwell time is 10 μs. **C** Representative images at different layers and time points from panels (**A**, **B**). The RPOC region of interest, selected via software, is outlined. APX (magenta) are automatically identified by RPOC at each axial layer and time point from mitochondria within the selected area.

One of the key advantages of RPOC is its ability to enable real-time, site-specific control of molecular activities in live cells. It offers unprecedented spatial precision for targeting structures within a 3D volume, particularly when using fs laser pulses^108^. RPOC can automatically select targets based on optical signals and track highly mobile structures without prior knowledge of the sample^108^. Figure 6 illustrates an example of RPOC performing microsurgery on highly dynamic mitochondria in live HeLa cells in 3D. First, an ROI is defined using the RPOC software, outlining a specific cell within a 3D population (Fig. 6C). Cells are labeled with MitoTracker Red, enabling the identification of mitochondria through fluorescence excitation using 473 nm or 589 nm lasers (Fig. 6A). Next, 3D time-lapse RPOC is conducted using a 1045 nm fs laser to selectively perturb mitochondria within the designated cell.

APXs are automatically selected based on MitoTracker signals at each axial plane and time point (Fig. 6B). These APX locations change dynamically in every frame due to mitochondrial mobility and fluctuations in MitoTracker signals. Four spatiotemporal frames of MitoTracker images and their corresponding APX are selected and displayed in Fig. 5C. The results demonstrate that fs laser pulses selectively interact with only mitochondria within the targeted cell and layer in 3D.

A comparative 3D visualization of MitoTracker signals before and after RPOC is provided in Supplementary Videos S1,2. These results showcase the capability of RPOC to achieve real-time, highly precise photo-perturbation of mobile targets in 3D within live cells.

### Controlling states of photochromic molecules

**Fig. 7:**
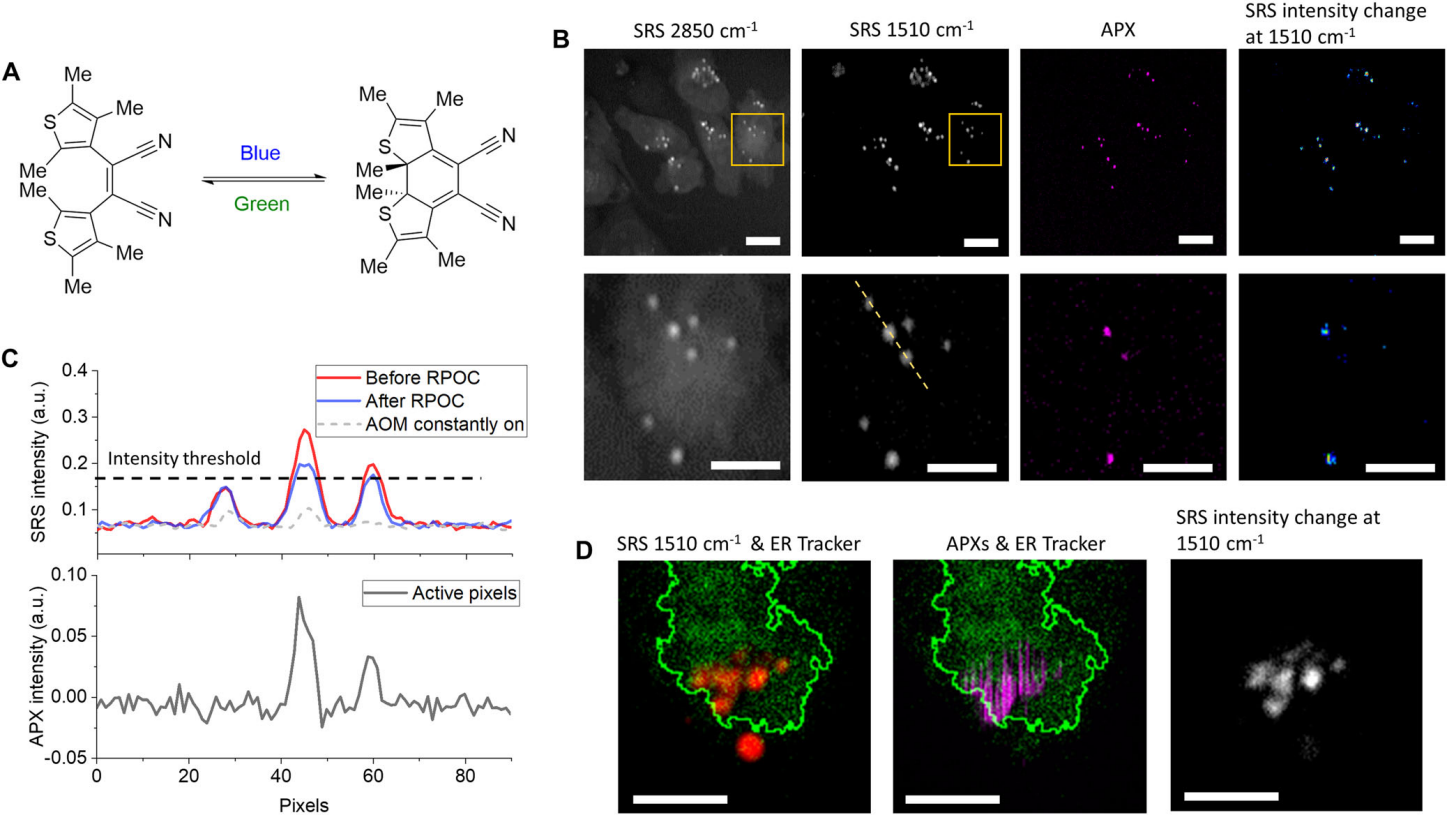
RPOC-controlled site-specific state transition of photochromic molecules visualized by stimulated Raman scattering (SRS) microscopy. **A** Molecular structures and photoconversion of *cis* − 1,2-dicyano1,2-bis(2,4,5-trimethyl-3-thienyl)ethene (CMTE). **B** SRS images at different Raman transitions, active pixels (APXs), and the SRS intensity changes for cells treated with CMTE. The bottom rows show enlarged areas of the selected regions in the top row. CMTE is treated using only the 532 nm laser at the selected APXs. Scale bars: 10 µm for top panels, 5 µm for bottom panels. **C** SRS intensity of the CMTE signals before and after green light treatment, and the APX intensity along the selected line in panel (**B**). **D** SRS signals at 1510 cm^−1^ for CMTE (red), endoplasmic reticulum (ER) signals from ER-Tracker (green), and APX for the 532 nm laser treatment (magenta). The ER boundary is outlined in green curves. The SRS intensity change at 1510 cm^−1^ shows where the CMTEs are converted to the open form in the cell. Adapted with permission from ref. ^46^ Scale bars: 5 µm.

The first RPOC platform was developed using a stimulated Raman scattering (SRS) microscope^46^. In this system, target selection is based on SRS signal detection, which measures Raman transitions of molecules at specific wavenumbers. This RPOC system has demonstrated precise control over the state of photochromic molecules at only desired locations. For example, we have performed precise control of the state of *cis* − 1,2-dicyano1,2-bis(2,4,5-trimethyl-3-thienyl)ethene (CMTE) in subcellular areas using CMTE signals or ER signals as discriminators^46,103^. This molecule, when illuminated with a 405 nm laser, switches to a closed form that exhibits a strong Raman transition at 1510 cm^−1^ (Fig. 7A)^46^. Conversely, when illuminated with a 532 nm laser, it changes to an open-*cis* form devoid of this peak^46^. By setting a threshold using a comparator circuit, RPOC can selectively interact with aggregates exhibiting SRS signals above the intensity threshold, leaving aggregates with lower signals untouched (Fig. 7B, C)^103^. The spatial precision is on the level of 500 nm. Additionally, RPOC can automatically convert CMTE accumulated in the ER to the open form using ER-Tracker signals while maintaining other aggregates in the closed form (Fig. 7D)^46^.

### Activating photoswitchable inhibitors at desired locations

**Fig. 8:**
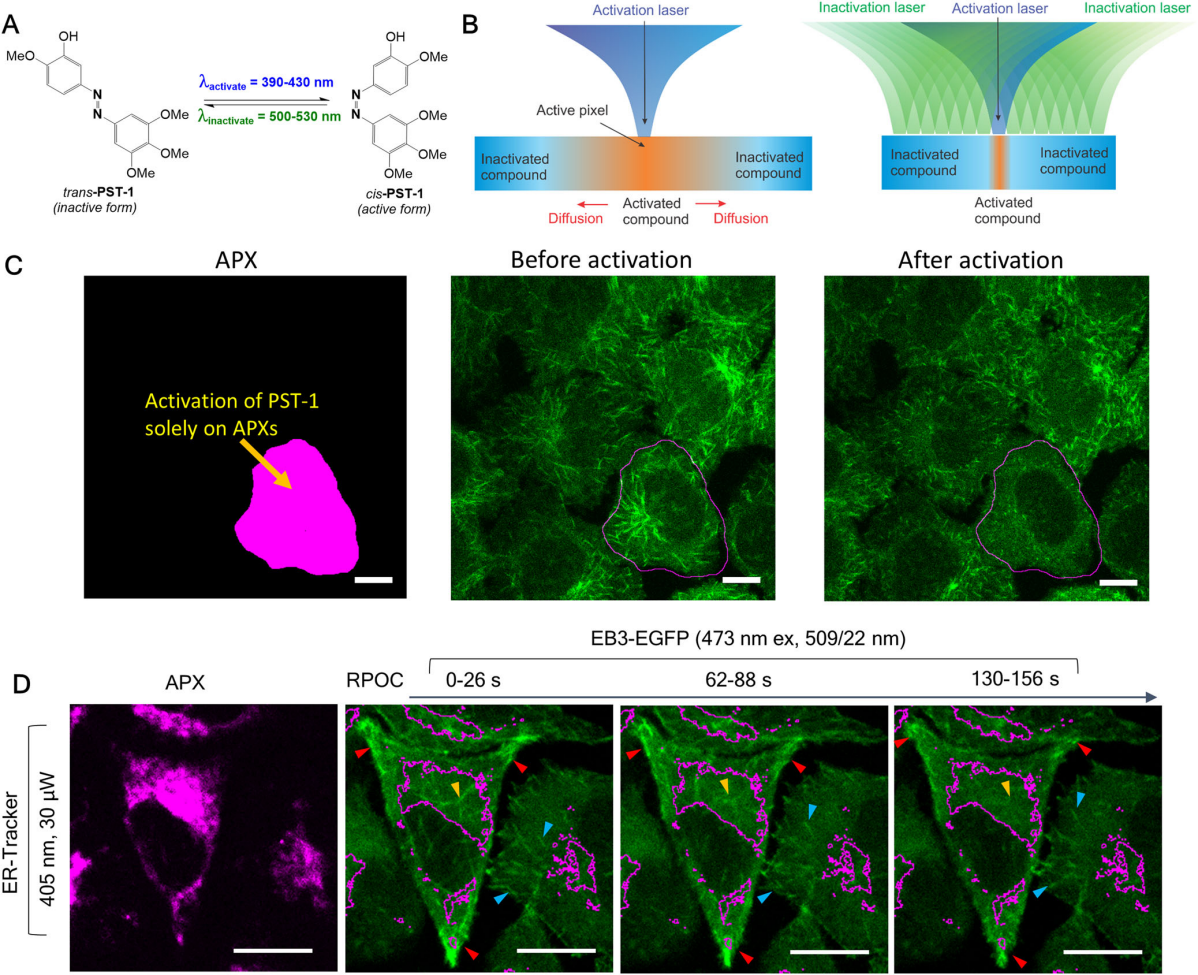
Selective activation of a photoswitchable microtubule polymerization inhibitor at single-cell and subcellular levels using RPOC. **A** Molecular structure and light conversion properties of PST-1. **B** A schematic comparing the use of blue light alone for the activation of photoswitchable compounds versus the use of both blue and green light for spatial confinement of the activated compound. The latter allows for inactivation of the compound once diffused out of the desired active pixels (APXs). **C** APXs (magenta) for PST-1 activation and EB3-EGFP signals (green) before and after selective PST-1 activation only in the selected cell. Scale bars: 10 µm. **D** APXs (magenta) and selective inhibition of microtubule polymerization on the endoplasmic reticulum (ER). The ER is labeled with ER-Tracker and outlined (magenta) in the EB3-EGFP (green) images. A 405 nm laser is used for PST-1 activation. The EB3-EGFP signals are excited using a 473 nm laser. Panels (**A**, **B**, **D**) adapted with permission from ref. ^48^ Scale bars: 20 µm.

In addition to controlling the state of photochromic Raman-active molecules, RPOC can selectively activate photoswitchable inhibitors in specific cells or subcellular regions, enabling precise, site-specific control of biomolecular activities. Conventional methods of using inhibitors involve directly adding them to the cell culture, relying on the chemical’s specificity for the cells of interest. However, chemical-based specificity often falls short, resulting in off-target effects. RPOC provides higher spatiotemporal control over the reactivity of inhibitors if they can be rendered photoresponsive. Inhibitors can be photocaged or coupled with photoswitches to create active and inactive states^25,106,109^. The photoswitchable inhibitor is administered in its inactive form, followed by localized activation at desired targets. An example of this approach is the site-specific activation of a photostatin PST-1, a photoswitchable inhibitor of microtubule (MT) polymerization (Fig. 8A)^25,46^. Activation was achieved by illuminating target sites with the activation laser while using the inactivation laser elsewhere (Fig. 8B)^48^. This ensures that even if the activated inhibitor diffuses to nearby locations, it will be instantly rendered inactive. Even without the inactivation laser, it was observed that only the selected cell, where PST-1 was activated at the APXs, showed a significant decrease in MT polymerization dynamics, whereas adjacent cells did not (Fig. 8C)^107^. This MT polymerization inhibition can also be achieved at the subcellular organelle level, such as the ER (Fig. 8D)^48^. Using this approach, RPOC can be paired with other photoswitchable inhibitors to manipulate local chemical processes. Another potential application is using photoswitchable actin inhibitors like neo-optojasp and optolat with RPOC to precisely control actin polymerization in subcellular areas^106,109^. Controlling biochemical processes with such spatiotemporal precision provides valuable insights into site-specific biochemical influences on cell functions and enhances our understanding of compound interactions with heterogeneous cells and subcellular compartments.

### Generating ROS or LDP in selected organelles

Another direct function of the action laser is the induction of ROS using blue lasers. Short-wavelength light interacts with cellular chromophores like flavins and cytochromes, promoting them to higher energy states^110^. The excited state energy can then be transferred to molecular oxygen and therefore produce ROS. The generated ROS creates oxidative stress in cells, impacting various cellular pathways. The nature and extent of damage vary based on the site of perturbation within cells. For instance, photodynamic therapy targeting the ER region has been reported to be more effective than other organelle-specific photosensitizations^111^. Using RPOC technology, the impact of localized exposure to blue light can be monitored with organelle-level precision^48^. Interactions of blue light with mitochondria and ER led to significantly greater disruption of MT polymerization dynamics compared to interactions with the nucleus or lipid droplets^48^. RPOC allows precise control of laser dosage on selected organelles, correlating with cellular responses. For example, a higher dosage of the 405 nm laser on ER induces a faster decrease of fluorescence signals from enhanced green fluorescence protein (EGFP) conjugated with end binding protein 3 (EB3) (EB3-EGFP) in HeLa cells^48^. The rapid decay of the MT signal is likely due to a higher level of ROS generated on mitochondria or ER. The H_2_O_2_ produced at APX can possibly diffuse out and oxidize the EBG-EGFP molecules. The newly developed software-assisted RPOC even permits outlining any targeted cell and selectively perturbing desired cellular organelles within this cell^107^. This capability allows visualization of both the EGFP signal loss induced by ROS generation in mitochondria and the leakage of MitoTracker into the cytosol due to mitochondrial damage^107^.

When fs laser pulses with high peak power are used, RPOC can also induce low-density plasma (LDP) at targeted locations^108^. This LDP is generated through multiphoton absorption, making it significantly more localized to the laser focus compared to CW-laser-based linear interactions^108^.

## Discussion and perspectives

Optical control methods, including nonspecific illumination, PI, point scanning, and RPOC, each have distinct strengths and weaknesses, making them suitable for different applications. When large-area treatment is required at the tissue level, nonspecific illumination has unique advantages. It covers a wide sample area, has minimal technical complexity, and can penetrate deep into biological tissues. PI can also be used in these cases, but it is limited to treating only surface tissue areas because optical scattering within tissues can distort the illumination pattern, reducing spatial accuracy. However, PI is particularly beneficial when simultaneous, continuous treatment of multiple surface targets is needed, such as in controlling neurons with optogenetics.

Point scanning and RPOC offer much higher spatial precision compared to nonspecific illumination and PI, but are currently mostly applied in a relatively small FOV. These technologies can be scaled up to treat areas from millimeters to meters, but with reduced spatial accuracy. When applied in microscopic setups, point scanning and RPOC provide high 3D sectioning capabilities and spatial precision that both global and PI cannot achieve. Moreover, compared to PI, RPOC is capable of handling highly dynamic, mobile targets and can simultaneously apply action lasers across a broad wavelength range, from UV to IR. Currently, point scanning and RPOC are mostly applied for microscopic applications, advancing a fundamental understanding of cell functions and drug activities by controlling site-specific chemical processes in live cells and small organisms.

The closed-loop feedback control system has been widely used for both large-scale and microscale optical control of animal behaviors^112^, cell responses^72^, and particle movements^74,78^. RPOC used the same concept and tailored it for high-speed laser scanning microscopy, chemical detection, and real-time laser activation. Compared to conventional point-scanning and PI methods, RPOC offers unique capabilities, particularly in automated and real-time laser activation based on chemical-specific optical signals. It can precisely track and simultaneously treat mobile targets with nanosecond response time without prior knowledge of the sample. Compared to traditional point-scanning methods for light delivery, RPOC usually reduces phototoxicity due to the utilization of short pixel dwell times and targeted illumination, limiting exposure to only the desired molecular entities. Compared to PI methods, the potential phototoxicity might be higher due to the tightly focused laser spot.

From a technological perspective, developing a more compact and user-friendly platform would further enhance the applicability of RPOC in the biological field. Currently, fs laser-based 3D precision perturbation of cellular compartments has just been demonstrated with RPOC^108^. Further studies, particularly those exploring the wavelength and peak power dependence of cellular perturbation, could provide deeper insights into the light-matter interaction mechanisms underlying these opto-control processes. Fs lasers can also be applied to uncage molecules or ions using the IR wavelength through two-photon uncaging^33,113^. Additionally, the use of NIR or mid-IR lasers, which primarily induce local heating^114^, has not yet been explored with RPOC. Investigating this approach could improve our understanding of how localized temperature increases influence cellular behavior. On the imaging front, integrating label-free contrast mechanisms such as harmonic generation, CRS, and IR absorption could introduce new modalities for target selection and readout, further expanding the capabilities of RPOC.

Current RPOC systems utilize one set of 2D galvo mirrors for raster scanning of combined lasers for excitation, readout, and action lasers. To enable more complex opto-control that might require different pixel dwell time than imaging, an additional set of galvo mirrors can be employed. Furthermore, RPOC can be integrated with wide-field illumination and high-speed imaging using fast cameras.

The development of more photoswitchable or photolabile compounds to pair with RPOC can enhance the precise control of cellular or subcellular molecular activities. When used with RPOC, these photoactive chemicals can be precisely activated and confined to desired subcellular locations. This capability would enable a better understanding of the site-specific functionality of drugs and biomolecules.

Beyond its applications in single cells, RPOC shows great promise for enabling precise control of single-cell behaviors in 3D organoids, tissue samples, and live animals. For in vivo applications, RPOC offers advantages when both high spatial resolution and precision in 3D are required, as it is intrinsically integrated with multiphoton excitation fluorescence and confocal fluorescence microscopy. This integration only requires the addition of a few optoelectronic components to any existing microscope, giving the capability to simultaneous control and imaging of entities in deeper tissue layers. In contrast to PI methods, which generally lack the 3D sectioning capability or exhibit reduced performance in spatial resolution, RPOC provides more precise control over deeper tissue layers and generates superior image quality for target selection and readout recording.

RPOC has the potential to scale up and be applied in laser treatment and ablation of tissue samples. Feedback-controlled laser ablation methods have been developed to ensure precise control over the thermal effects on tissues, minimizing unwanted damage. The most common feedback mechanisms involve temperature sensors that provide real-time feedback to adjust laser settings (e.g., power, duration)^115^. This strategy regulates treatment duration and prevents excessive thermal damage, offering a more adaptive and responsive approach compared to traditional fixed-setting laser ablation. RPOC would further advance these methods by enabling high-speed and pixel-based decision-making, providing chemical contrasts for target selection, and facilitating deeper tissue treatment and ablation using multiphoton processes.

## Supplementary information

Supplementary information

Supplementary video1

Supplementary video2

## Data Availability

Raw data related to Fig. 6 are deposited in Figshare: 10.6084/m9.figshare.28510247. (https://figshare.com/s/f7b5ec8955ff66fd6547).
