# Supplementary material for: Advances and future trends in real-time precision optical control of chemical processes in live cells
Source: Npj Imaging. 2025 May 28;3:23. doi: 10.1038/s44303-025-00083-1 (PMC12119352; doi:10.1038/s44303-025-00083-1)
Supplement: Supplementary file 1 — Supplementary information [file 44303_2025_83_MOESM1_ESM.pdf]

Supplementary information

Advances and Future Trends in Real-Time Precision Optical Control of Chemical Processes in Live Cells

Authors: Chi Zhang[1,2,3]*, Bin Dong[1,2], Shivam Mahapatra[1,2], Seohee Ma[1,2]

[1]James Tarpo Jr. and Margaret Tarpo Department of Chemistry, Purdue University, 560 Oval Dr., West Lafayette, IN 47907, USA
[2]Purdue Institute for Cancer Research, 201 S. University St., West Lafayette, IN 47907, USA.
[3]Purdue Institute of Inflammation, Immunology, and Infectious Disease, 207 S. Martin Jischke Dr., West Lafayette, IN 47907, USA.
*zhan2017@purdue.edu

Supplementary Video captions

Supplementary Video 1. Depth-resolved and time-lapse images of MitoTracker signals during RPOC. The treated cell is indicated in Figure 6.

Supplementary Video 2. Reconstructed 3D time-lapse images of MitoTracker signals during RPOC. The treated cell is indicated in Figure 6.
